# The Overlooked Aesthetic Function: The Impact of Food Anthropomorphism on Taste Perception

**DOI:** 10.3390/foods15061020

**Published:** 2026-03-14

**Authors:** Siyue Zhang, Kai He, Lexin Su, Yuanxin Hu, Hao Hong, Siyuan He, Yun Liu, Xinyi Ni, Fuqun Liang, Wenxuan Liu, Dan Huo, Chenjing Wu

**Affiliations:** 1 College of Economics and Management, South China Agricultural University, Guangzhou 510642, China; siyue.zhang@psy.ox.ac.uk (S.Z.); hekaiclire@scau.edu.cn (K.H.); 2 School of Psychology, South China Normal University, Guangzhou 510631, China; 2022023822@m.scnu.edu.cn (L.S.); 2023023785@m.scnu.edu.cn (Y.H.); 2024024027@m.scnu.edu.cn (H.H.); 13688955287@163.com (Y.L.); 2024024120@m.scnu.edu.cn (X.N.); liangfuqun523@163.com (F.L.); liuwenxuan1115@163.com (W.L.); 3 Department of Experimental Psychology, University of Oxford, Oxford OX1 2JD, UK; 4 Div of Psychology & Lang Sciences, University College London, London WC1E 6BT, UK; nick.he.23@ucl.ac.uk; 5 School of Psychology and Entrepreneurship, Guangdong University of Finance, Guangzhou 510521, China; 6 Faculty of Psychology, Beijing Normal University, Beijing 100875, China; 7 School of Environment, Liaoning Unversity, Shenyang 110036, China

**Keywords:** anthropomorphism, aesthetics, gustatory perception, food consumption

## Abstract

Anthropomorphism is a commonly used strategy in food marketing; however, its effect on perceived taste remains controversial. Moreover, a frequently overlooked issue is that anthropomorphic designs often alter the aesthetic appeal of food. Accordingly, the present research study focused on the joint effects of anthropomorphic strategies and food aesthetics on taste perception. Across four studies, these effects were examined at different stages of consumption. Study 1 employed an online study and demonstrated that at the food choice stage, anthropomorphism exerted a positive effect on perceived taste. Building on this design, Study 2 varied the type of food and introduced consumption cues in an online context, and the results showed that anthropomorphism did not exert a significant negative effect on taste perception. In contrast, aesthetic appeal consistently and positively predicted taste evaluations across conditions. Study 3 used a simulated eating task while controlling for aesthetic factors, and the results indicated that after consumption, anthropomorphism negatively affected taste perception. Finally, Study 4 investigated actual eating behavior and showed that in delayed evaluation contexts, anthropomorphic strategies reduced consumers’ taste evaluations when aesthetic appeal was held constant. More critically, anthropomorphism has a beneficial effect at the food choice stage but elicits negative taste perceptions following actual consumption. Furthermore, when aesthetic factors are controlled, anthropomorphism consistently lowers taste evaluations in delayed evaluation contexts.

## 1. Introduction

Anthropomorphism, a commonly used tactic in food marketing [1], refers to the tendency to attribute human characteristics to nonhuman entities [2,3,4,5]. At present, food marketers frequently employ anthropomorphic strategies to promote food products [6,7,8,9]. The findings from a substantial body of research on anthropomorphic food marketing suggest that anthropomorphism can elicit favorable consumer evaluations of food [10,11]. However, the authors of other studies indicate that anthropomorphism may exert negative effects on food choice [12,13,14]. For example, Cooremans and Geuens [10] found that compared with non-anthropomorphized fruits and vegetables, consumers perceived those that were misshapen and anthropomorphized to be better-tasting. In contrast, Schroll et al. [15] reported that when consumers compared anthropomorphized and non-anthropomorphized apples, they expected to have a lower level of enjoyment from eating the former.

In addition, with growing market competition and societal development, researchers have become increasingly aware of the importance of aesthetic factors during both the food choice and consumption stages [16,17]. In recent years, aesthetics have emerged as a key dimension in consumers’ evaluations of food [18,19]; prior research has shown that visually appealing foods can evoke more positive sensory experiences (e.g., higher perceived tastiness) and stimulate stronger consumer responses [20,21,22].

However, what is often overlooked is that when anthropomorphic marketing strategies are applied to food, the aesthetic attributes of the food itself may also be altered. As noted by Cooremans and Geuens [10], anthropomorphism provides a means of mitigating negative taste perceptions that arise from unattractive appearances. Importantly, anthropomorphized food products that gain popularity in the marketplace typically possess high aesthetic appeal, and consumers tend to hold more favorable attitudes toward foods with more attractive appearances [23]. A critical gap in the existing literature is that prior research on food anthropomorphism has not accounted for potential aesthetic differences between anthropomorphized and non-anthropomorphized foods. For instance, the aesthetic appeal of misshapen vegetables may increase after anthropomorphization, yet this possibility has not been considered in comparison with non-anthropomorphized misshapen vegetables.

In the present article, we argue that neglecting the role of food aesthetics in prior research may have led to the conclusion that the positive effects attributed to food anthropomorphism are, in essence, driven by concomitant increases in aesthetic appeal resulting from anthropomorphic design. Theoretically, by disentangling and controlling the effects of both anthropomorphism and visual aesthetics on food evaluations, through the current research study, we will address an important gap in the existing literature.

Moreover, in the present research study, we employ multiple experimental methodologies, including simulated eating tasks [24] and actual consumption paradigms [25], both of which have been validated as meaningful approaches in prior research. We are therefore better able to capture discrepancies between consumers’ anticipatory evaluations and their actual tasting experiences, particularly with respect to the effects of food anthropomorphism when visual aesthetics are held constant. In addition, we further examine the context of repeated anthropomorphized food evaluation, thereby extending the literature scope by elucidating how prior experiences with anthropomorphized foods influence consumers’ short-term food choice behavior.

From a practical perspective, in the present research study, we highlight the importance of incorporating aesthetic considerations when evaluating the effects of food anthropomorphism on consumer behavior. Given that anthropomorphized foods are widely used in the food marketplace, particularly in retail contexts, marketers must carefully examine the interplay between anthropomorphic strategies and food aesthetics, as well as strategically deploy anthropomorphism in appropriate contexts to avoid unintended negative consequences.

By disentangling and controlling the effects of anthropomorphism and visual aesthetics on food evaluations, in the current research study, we theoretically address an important gap in the existing literature. Moreover, while prior studies have documented discrepancies between expected and actual taste experiences, we are the first to systematically investigate how this discrepancy is influenced by the interplay of anthropomorphism and aesthetics across distinct consumption stages (choice, simulated consumption, actual consumption, and repeated evaluation). We propose a stage-dependent model where anthropomorphism’s effect reverses from positive (at the choice stage) to negative (post-consumption) when aesthetic appeal is held constant.

## 2. Literature Review

### 2.1. Anthropomorphic Strategies and the Aesthetic Appeal of Food

As early as 1936, Apicius famously remarked that “we eat first with our eyes.” The close association between visual aesthetics and food consumption and evaluation has been extensively examined in both research and practice over recent decades. Empirical evidence demonstrates that attributes such as a food’s color and shape, even the shape of the plate on which it is served, can systematically influence consumers’ expectations of its taste [26,27,28].

Anthropomorphism is defined as the tendency to attribute human characteristics to nonhuman entities [2]. On the one hand, anthropomorphizing food has become a widely used marketing tactic, and prior research suggests that its effectiveness stems from anthropomorphism functioning as an inductive reasoning process, whereby consumers ascribe human-like mental capacities to nonhuman entities [2]. The more closely a nonhuman object’s appearance or behavior resembles that of humans, the more likely consumers are to anthropomorphize it and subsequently draw inferences about its unobservable attributes by relying on their self-knowledge [29]. As previous studies have shown, once foods are anthropomorphized, they can elicit a positive effect on consumers [30], which in turn increases both preference for these foods and their actual consumption.

On the other hand, with ongoing socioeconomic development, people have become increasingly attentive to the experiential aspects of eating. Food consumption is no longer merely a means of survival but has evolved into a multisensory and holistic experience [31,32,33]. Accordingly, consumers have grown more concerned with the visual aesthetics of food, and scholarly attention to food’s aesthetic appeal has likewise increased. Prior research indicates that consumers tend to perceive foods with greater aesthetic appeal as being more palatable [34] and healthier [24].

It is important to note that the application of anthropomorphic strategies to food is often accompanied by modifications to the food’s appearance, and in many contexts, this enhances its aesthetic appeal. Neglecting the aesthetic consequences of anthropomorphism has prevented researchers from fully uncovering the true impact of anthropomorphic strategies on consumers. As Cooremans and Geuens [10] suggested, anthropomorphism may exert stronger effects on foods with low initial aesthetic appeal—likely because anthropomorphic manipulation simultaneously and substantially increases visual attractiveness. Consequently, when aesthetic factors are adequately controlled, the effect of anthropomorphism itself may no longer be significant.

However, in real-world contexts, food choice and consumption are not simply one-off behaviors. Consumers’ evaluations of food may differ across stages, ranging from anticipatory judgments to actual consumption. Accordingly, the roles of aesthetics and anthropomorphism should be considered in a stage-specific manner across these phases of the consumption process.

### 2.2. Conceptual Framework: Why Effects Differ Across Consumption Stages

In prior research, anthropomorphism is often regarded as a “universal” strategy. However, by distinguishing between the choice and consumption stages, we find that anthropomorphism has different effects on food choice depending on stage. At the choice stage, anthropomorphized foods may appear to be warmer and friendlier and have a stronger sense of social presence [6,7,8,9], thereby increasing taste expectations.

Anthropomorphism may strengthen mental perception. At the consumption stage, when the act of “eating” becomes salient, one’s mental perception will elicit anticipated guilt about harming a “mind-bearing entity,” thereby reducing one’s enjoyment and willingness to eat the food. This conflict may not be salient at the earlier choice stage, but it is clearly amplified at the actual consumption stage.

However, more crucially, anthropomorphic manipulations often change the visual aesthetics of food. Aesthetics constitute an independent factor that has been fully demonstrated to drive taste expectations and enjoyment. Therefore, in order to identify the psychological role of anthropomorphism itself, we treat aesthetics as a theoretically distinct pathway that needs to be measured and controlled.

Overall, through the theoretical framework of this research study, we predict that anthropomorphism at the choice stage is more likely to increase taste expectations and choice preferences by enhancing warmth/social presence and positive emotions, whereas in the stage prior to consumption or during actual tasting, anthropomorphism is more likely to reduce taste evaluations by enhancing mental perception and eliciting moral concerns (such as guilt and discomfort).

Further, anthropomorphic cues may foster a stable mental representation, and the mental perception activated in the stage prior to consumption is not necessarily a one-off event. Even when separated temporally, when consumers are presented with the same anthropomorphized food again (e.g., in a secondary consumption context), anthropomorphism may continue to weaken taste-related evaluations and intentions through the mental perception and moral concern pathways.

### 2.3. Effect of Anthropomorphic Strategies on Food Choice Stage When Aesthetic Appeal Is Controlled

In the present research, the choice stage refers to the point at which consumers’ anticipatory evaluations and choice preferences are formed upon viewing food, prior to any actual tasting experience [35].

Zhao, Zhou, and Kang [30] examined the effects of anthropomorphic strategies on consumers’ preferences for and expectations of healthy foods, showing that anthropomorphizing foods (e.g., by assigning names or human-like facial expressions) significantly increased consumers’ preferences and purchasing intentions for these items while also eliciting a more positive affect. Janjić et al. [36] compared the effects of food images with high versus low visual aesthetic appeal on consumers’ taste expectations, health perceptions, and choice preferences, indicating that individuals were more likely to anticipate more visually appealing foods to be tastier and healthier and that these foods generated stronger choice intentions.

Synthesizing these findings and drawing on the theories of mind attribution and affective priming [2,30], we propose that at the initial choice stage, where consumption is not salient, anthropomorphism primarily operates by enhancing the food’s perceived warmth and relatability, leading to more positive inferences about its qualities, including taste. This effect is hypothesized to be independent of visual aesthetics when the latter is controlled.

Synthesizing these findings, we propose the following hypothesis:

**H1:**  *At the choice stage, anthropomorphic strategies exert a positive effect on consumers’ food perceptions when food aesthetics are controlled.*

### 2.4. The Effect of Anthropomorphic Strategies on Taste Perception During the Consumption Stage When Aesthetic Appeal Is Controlled

In the present research study, the consumption (tasting) stage refers to the point at which consumers evaluate the food and develop their subsequent purchase preferences after actual consumption.

Importantly, Schroll et al. [15] found that although consumers tend to prefer anthropomorphized foods at the choice stage, they often form negative evaluations when they realize that the anthropomorphized food will be physically consumed. This pattern suggests that the effects of anthropomorphism differ between the choice and actual consumption stages. Such differences arise because consumption is an intentional act that necessarily entails damage to the food. When a food is anthropomorphized, the harm caused by consumption may be interpreted as inflicting suffering on a “mind-bearing” entity.

With respect to food aesthetics, studies examining actual consumption have shown that more aesthetically appealing plating increases subjective ratings of flavor [37]. Integrating these findings, we propose the following hypothesis regarding consumers becoming aware that anthropomorphized foods will be destroyed through consumption:

**H2:**  *In actual tasting contexts, anthropomorphism reduces consumers’ evaluations of food when aesthetic appeal is controlled.*

Moreover, Schroll et al. [15] only examined the choice and post-consumption stages. Although their findings imply that anthropomorphized foods may influence eating experiences after subsequent repurchases, there is currently a lack of longitudinal evidence to support this proposition. Accordingly, in the present research study, we further investigate the effects of anthropomorphized foods on consumers’ eating experiences in repeated evaluation contexts while controlling for aesthetic appeal and ruling out potential aesthetic confounders.

We posit that during repeated evaluation, consumers may form negative evaluations of anthropomorphized foods in advance, leading to more unfavorable attitudes toward such products. Based on this reasoning, we propose the following hypothesis:

**H3:**  *When aesthetic appeal is controlled, consumers who initially consume anthropomorphized foods will experience more negative taste perceptions during secondary consumption than consumers who initially consume non-anthropomorphized foods.*

**Study**

**Hypothesis**

**Manipulation**

**Stage of Consumption**

**Dependent Variables**
1H12 (food type) × 2 (aesthetics)ChoiceGustatory Perception Eating Intention Purchasing Intention2H1, H22 (food type) × 2 (aesthetics)Choice and mild consumption cueGustatory Perception3H22 (food type) × 2 (consumption type)Simulated consumptionGustatory Perception Eating Intention Purchasing Intention4H2, H3Real tasting and 1-week follow-up 2 (food type) × 2 (consumption type)Actual and repeated evaluationBaseline Eating Intention and Hunger Level Gustatory Perception Eating Intention Purchasing Intention Repeated Evaluation

## 3. Study 1

Study 1 was designed to test H1 by examining the effects of anthropomorphism and aesthetics on taste expectations, eating intention, and purchasing intention at the choice stage—prior to any consumption cues. This study establishes a baseline for how anthropomorphism influences evaluations when consumption is not yet salient.

### 3.1. Study Design

This study employed a within-subjects design with two factors, namely, food type (anthropomorphic vs. non-anthropomorphic) and aesthetic appeal (high vs. low). The dependent variables encompassed gustatory perception, eating intention, and purchasing intention.

### 3.2. Participants

We collected data from a total of 128 participants. All participants were university students aged between 18 and 29 years (105 females; *M_age_* = 20.55, *SD_age_* = 2.31), and they received compensation for their participation. All procedures were sanctioned by the Institutional Review Board, and informed consent was obtained from all participants.

### 3.3. Food Materials

**Figure 1:**
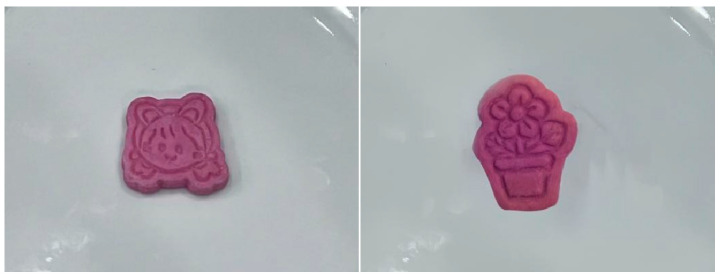

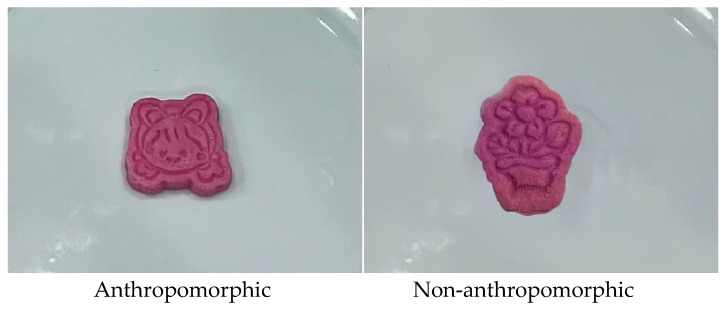
Different types of cookie images (food type and aesthetic appeal are different: the top shows high aesthetic, and the bottom shows low aesthetic).

This study utilized four distinct types of cookie images, which included anthropomorphic high aesthetic, anthropomorphic low aesthetic, non-anthropomorphic high aesthetic, and non-anthropomorphic low aesthetic (see Figure 1). All four cookie images maintained identical pixel dimensions of 1440 × 1920. Prior to the formal study, 36 participants who did not take part in the main study were recruited for material assessment (32 females; *M_age_* = 20.95, *SD_age_* = 2.63). They were instructed to evaluate the aesthetic appeal of the food depicted in the images. The results indicated a significant main effect of aesthetic appeal (*F*(1, 36) = 12.55, *p* = 0.001, *η_p_*^2^ = 0.26), demonstrating that irrespective of anthropomorphism, high-aesthetic cookies were perceived as more visually appealing than low-aesthetic cookies, thereby validating the effectiveness of the material selection.

### 3.4. Procedure

In this study, we disseminated participant recruitment advertisements via the WeChat platform. Upon seeing the recruitment information, potential participants contacted the research team, after which they were invited to the laboratory to partake in the field study.

Upon arriving at the laboratory, participants were instructed to complete an identical questionnaire that presented four different types of cookie images (anthropomorphic high aesthetic, anthropomorphic low aesthetic, non-anthropomorphic high aesthetic, and non-anthropomorphic low aesthetic). Participants were asked to adopt the perspective of a consumer, observing the images and contemplating the corresponding questions. The four types of cookie images were displayed in random order, and after viewing each image, participants were required to evaluate the cookies using a 9-point Likert scale (1 = not at all; 9 = very much) on various dimensions, including aesthetic appeal, gustatory perception, eating intention, and purchasing intention. Upon completing the observation and evaluation tasks, participants were further requested to provide demographic information, specifically regarding their gender and age.

For the evaluation of aesthetic appeal, participants were asked to assess “How beautiful does the appearance of the cookie look?”; for gustatory perception, they were instructed to evaluate “How tasty do you think this cookie would be?”; regarding eating intention, participants were requested to rate “How much do you desire to eat this cookie?”; and for purchasing intention, they were asked to consider “How much do you wish to buy this cookie?”

### 3.5. Results

We used SPSS 23.0 for Windows TM (IBM Corp., Armonk, NY, USA) to analyze the effects of food type and aesthetic appeal on the various dependent variables.

***Manipulation Check***. We conducted a Repeated Measures ANOVA to investigate the differences in aesthetic appeal among the four different types of cookies (see Table 1). The results indicated that the main effect of food type was not significant (*F*(1, 127) = 2.18, *p* = 0.14); the main effect of aesthetic appeal was significant (*F*(1, 127) = 52.45, *p* < 0.001, *η_p_*^2^ = 0.29). The interaction effect between food type and aesthetic appeal was not significant (*F*(1, 64) = 0.55, *p* = 0.82). Participants consistently rated high-aesthetic cookies as more visually appealing than low-aesthetic cookies, regardless of whether they were anthropomorphic or non-anthropomorphic. This finding demonstrates the effectiveness of our aesthetic manipulation.

**Figure 2:**
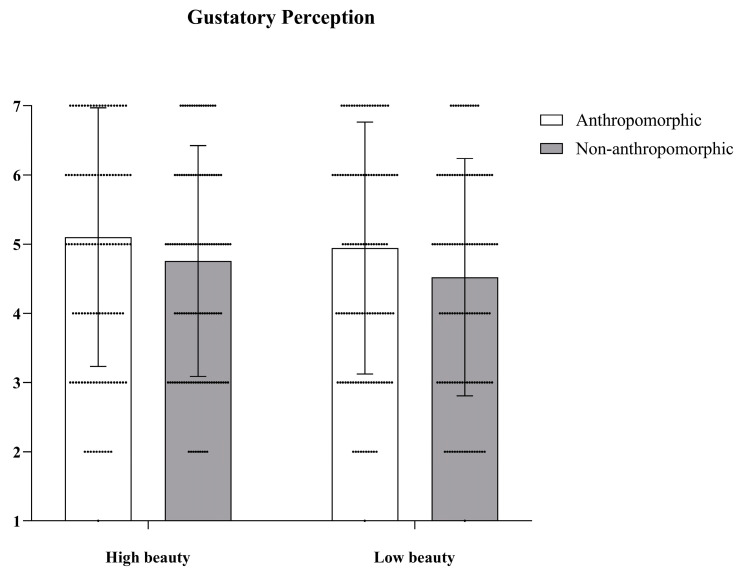
The effects of food type and aesthetic appeal on gustatory perception. The dots in the figure represent each participant’s data, the error bars indicate the standard error.

***Gustatory Perception.*** We conducted Repeated Measures ANOVA to examine differences in the gustatory perception of the four different types of cookies. The main effect of food type was significant, with *F*(1, 127) = 19.25, *p* < 0.001, *η_p_*^2^ = 0.13; the main effect of aesthetic appeal was also significant, with *F*(1, 127) = 10.76, *p* = 0.001, *η_p_*^2^ = 0.08; but their interaction effect was not significant, with *F*(1, 127) = 0.37, *p* = 0.54 (see Figure 2).

**Figure 3:**
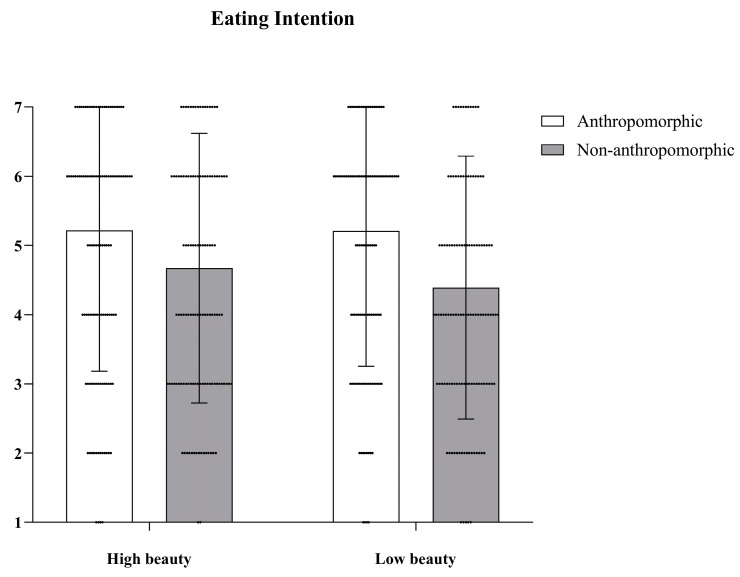
The effects of food type and aesthetic appeal on eating intention. The dots in the figure represent each participant’s data, the error bars indicate the standard error.

***Eating Intention.*** We conducted Repeated Measures ANOVA to examine differences in the eating intention of the four different types of cookies. The main effect of food type was significant, with *F*(1, 127) = 43.55, *p* < 0.001, *η_p_*^2^ = 0.26; the main effect of aesthetic appeal was also significant, with *F*(1, 127) = 4.27, *p* = 0.04, *η_p_*^2^ = 0.03; but their interaction effect was not significant, with *F*(1, 127) = 3.42, *p* = 0.07 (see Figure 3).

**Figure 4:**
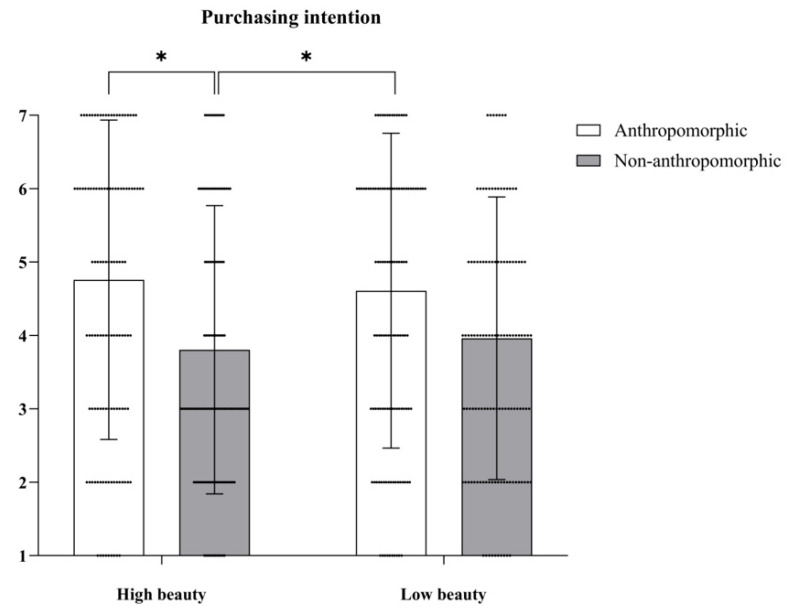
The effects of food type and aesthetic appeal on purchasing intention. The dots in the figure represent each participant’s data, the error bars indicate the standard error, and the asterisks denote statistical significance at *p* < 0.05.

***Purchasing Intention.*** We conducted a Repeated Measures ANOVA to assess differences in purchasing intention for the four different types of cookies. The main effect of food type was significant, with *F*(1, 64) = 56.45, *p* < 0.001, *η_p_*^2^ = 0.31; conversely, the main effect of aesthetic appeal was not significant, with *F*(1, 64) = 0.33, *p* = 0.94. Notably, the interaction effect between food type and aesthetic appeal was significant, with *F*(1, 64) = 5.41, *p* = 0.02, *η_p_*^2^ = 0.04 (see Figure 4). Further simple effects analysis revealed that participants exhibited a higher purchasing intention for anthropomorphic cookies. Specifically, the purchasing intention for anthropomorphic cookies with high aesthetic appeal was the highest (*ps.* < 0.05), while the purchasing intention score for anthropomorphic cookies with low aesthetic appeal was significantly greater than that for non-anthropomorphic cookies with high aesthetic appeal (*p* < 0.05).

The results of Study 1 were consistent with our expectations: In a context involving only the choice stage, both the aesthetic appeal of food and anthropomorphic strategies exerted significant positive effects on consumers’ gustatory perceptions and eating intentions. And there were no significant interactions between the aesthetic factors of food aesthetic appeal and anthropomorphism strategies in terms of gustatory perception and eating intention. Instead, only significant effects of both factors on purchasing intention were observed. Moreover, the anthropomorphism strategy effectively mitigated the negative influence of low aesthetic appeal on purchasing intention.

However, Study 1 was limited to a mere food evaluation without addressing actual eating behavior, raising concerns about ecological validity. Consequently, Study 2 will involve a larger sample size, utilizing a more ecologically valid experimental approach through imagined eating scenarios, and will employ new experimental materials to further explore the effects of anthropomorphism strategies and food aesthetic appeal on gustatory perception when cues indicating “food will be eaten” are introduced during the selection phase.

## 4. Study 2

Study 2 aimed to introduce a subtle cue that the food would be eaten (“hand-with-chopsticks”) while still remaining in a pre-consumption, imagination-based context. The goal was to preliminarily explore whether making consumption slightly more salient would begin to attenuate the positive effect of anthropomorphism (a precursor to H2) while continuing to control for aesthetics.

### 4.1. Study Design

This study employed a between-subjects design with two factors, namely, food type (anthropomorphic vs. non-anthropomorphic) and aesthetic appeal (high vs. low). The dependent variable was gustatory perception.

### 4.2. Participants

We collected data from a total of 226 participants. All participants were university students aged between 18 and 41 years (82 males; *M_age_* = 20.65, *SD_age_* = 2.74), and they received compensation for their participation. All procedures were sanctioned by the Institutional Review Board, and informed consent was obtained from all participants. All participants received monetary compensation (CNY 20) upon completion of the study.

### 4.3. Food Materials

**Figure 5:**
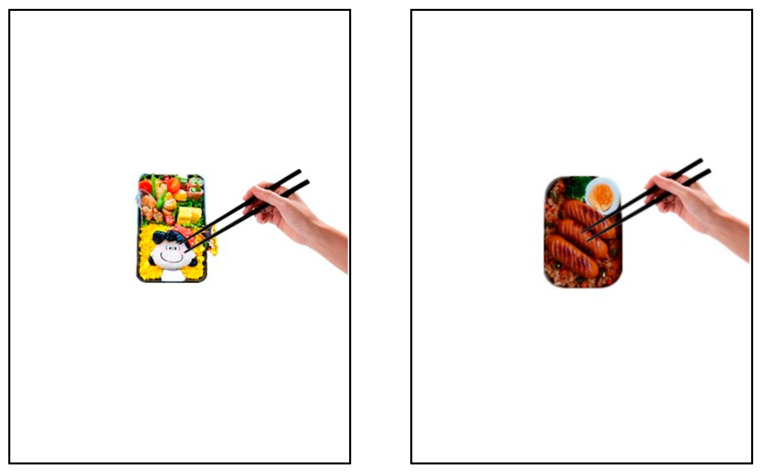

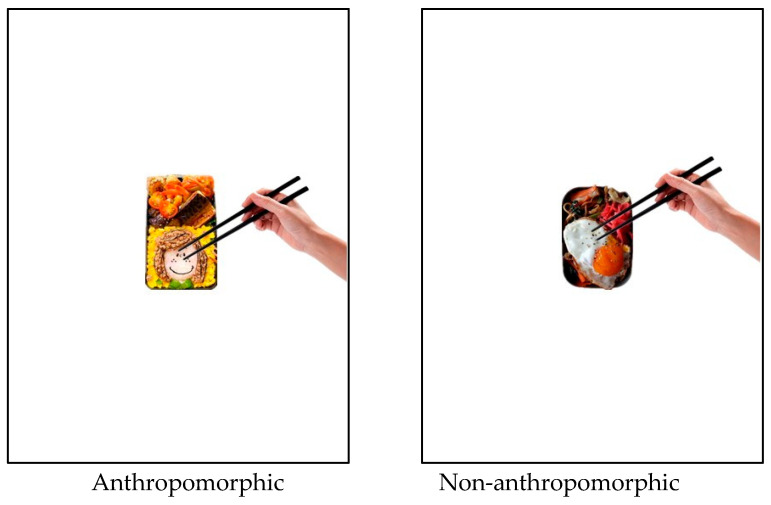
Different types of bento pictures (food type and aesthetic appeal are different: the top shows high aesthetic, and the bottom shows low aesthetic).

This study utilized four distinct types of bento images, including anthropomorphic high aesthetic, anthropomorphic low aesthetic, non-anthropomorphic high aesthetic, and non-anthropomorphic low aesthetic (see Figure 5). The image materials used in Study 2 were chosen from the public archive, available online at http://baidu.com/ (accessed on 16 December 2024). There are no conflicts of interest or ethical concerns involved. In the study, each image was presented to the participants in the same size, with a “food will be eaten” cue attached to each image. Specifically, the images depicted a hand holding chopsticks above the food, simulating the hand movements involved in eating. Prior to the main study, 28 participants who did not take part in the primary study (23 females; *M_age_* = 22.46, *SD_age_* = 2.35) were recruited to assess the materials. They were instructed to evaluate the aesthetic appeal of the food depicted in the images. Each participant was required to rate each type of bento image, completing a total of four evaluations. The results of a Repeated Measures ANOVA revealed a significant main effect of aesthetic appeal (*F*(1, 27) = 14.38, *p* = 0.001, *η_p_*^2^ = 0.35), whereas the main effect of food type and the interaction between aesthetic appeal and food type were not significant (*ps.* > 0.05). These results suggest that regardless of anthropomorphism, high-aesthetic food was perceived as more visually appealing than low-aesthetic food, thus validating the effectiveness of the material selection.

### 4.4. Procedure

Study 2 was conducted online. The researcher posted recruitment advertisements on an online platform, allowing interested individuals to contact the researcher and register for the study. Upon successful registration, participants were provided with the corresponding survey link. After completing the experimental tasks outlined in the questionnaire as instructed, participants received compensation for their participation.

Participants in the study were randomly assigned to one of four groups (anthropomorphic high aesthetic, anthropomorphic low aesthetic, non-anthropomorphic high aesthetic, and non-anthropomorphic low aesthetic) and were instructed to complete the corresponding questionnaire. Prior to the study, participants were asked to provide demographic information (gender and age) and report their current level of hunger to control for any potential effects of hunger on the study. Subsequently, participants were informed that they would be performing a food imagination tasting task, during which an image of a bento would be presented (see Figure 5). They were instructed to carefully observe the appearance of the food and imagine that the hand depicted in the image was their own, as if they were tasting the food. After completing the imagination eating task, participants were asked to rate the food’s gustatory perception on a 7-point scale (1–7, with higher numbers indicating greater deliciousness).

### 4.5. Results

***Manipulation Check***. We conducted a two-factor analysis of variance (ANOVA) to investigate the differences in aesthetic appeal among the four different types of bento (see Table 2). The results indicated that the main effect of food type was not significant (*F*(1, 222) = 2.17, *p* = 0.14); the main effect of aesthetic appeal was significant (*F*(1, 222) = 56.83, *p* < 0.001, *η_p_*^2^ = 0.20). The interaction effect between food type and aesthetic appeal was not significant (*F*(1, 222) = 2.45, *p* = 0.12). Participants consistently rated high-aesthetic bento as more visually appealing than low-aesthetic bento, regardless of whether they were anthropomorphic or non-anthropomorphic. This finding demonstrates the effectiveness of our aesthetic manipulation.

We also conducted a two-factor analysis of variance (ANOVA) to investigate the differences in Anthropomorphism Rating among the four different types of bento (see Table 3). The results indicated that the main effect of food type was significant (*F*(1, 222) = 371.57, *p* < 0.001, *η_p_*^2^ = 0.63); the main effect of aesthetic appeal was not significant (*F*(1, 222) = 0.03, *p* = 0.87). The interaction effect between food type and aesthetic appeal was not significant (*F*(1, 222) = 1.76, *p* = 0.008). Participants consistently rated the anthropomorphic bento used in the experimental design as appearing more human-like than the non-anthropomorphic bento, regardless of whether they were high in aesthetic appeal or low in aesthetic appeal. This finding demonstrates the effectiveness of our manipulation of the bento’s level of anthropomorphism.

***Hunger level.*** We conducted a two-factor analysis of variance (ANOVA) to examine the differences in hunger levels among the four different types of foods (see Table 4). The results indicated that the main effect of food type was not significant (*F*(1, 222) = 1.00, *p* = 0.32), the main effect of aesthetic appeal was also not significant (*F*(1, 222) = 0.09, *p* = 0.76), and the interaction between food type and aesthetic appeal was also not significant (*F*(1, 222) = 0.26, *p* = 0.61). These results suggest that there were no significant differences in hunger levels between the groups; thus, hunger level was not be considered as a potential confounding factor in subsequent analyses.

**Figure 6:**
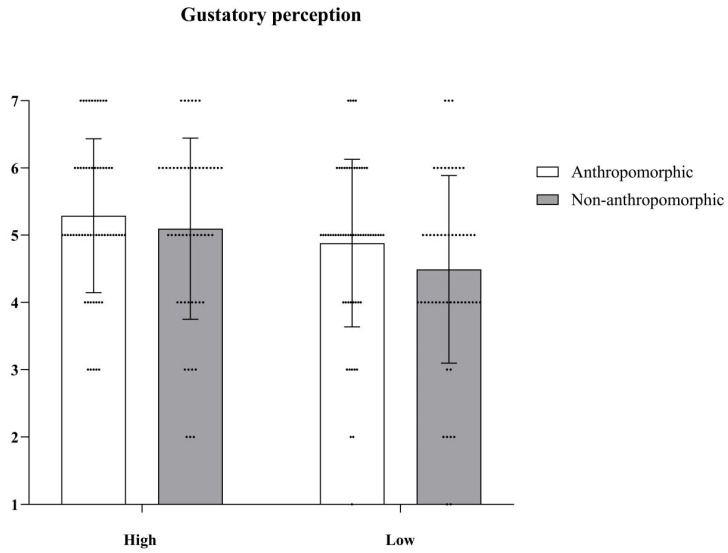
The effects of food type and aesthetic appeal on gustatory perception. The dots in the figure represent each participant’s data, the error bars indicate the standard error.

***Gustatory Perception.*** We conducted a two-factor analysis of variance (ANOVA) to examine differences in the gustatory perception of four different types of foods. The main effect of food type was not significant, *F*(1, 222) = 2.82, *p* = 0.10; the main effect of aesthetic appeal was significant, *F*(1, 222) = 9.12, *p* < 0.001, *η_p_*^2^ = 0.04; but their interaction effect was not significant, *F*(1, 222) = 0.39, *p* = 0.53 (see Figure 6).

The results of Study 2 were not entirely consistent with H2, as we did not observe a significant negative effect of the anthropomorphism strategy on gustatory perception. Instead, we found a significant positive effect of the aesthetic factors of food aesthetic appeal on gustatory perception. However, the inconsistency between the two studies may stem from the presence or absence of the “food will be eaten” cue. We hypothesize that when the cue “food will be eaten” is present, indicating food tasting, the anthropomorphism strategy does not have a positive effect on gustatory expectations. The non-significant results in Study 2 may be attributed to the insufficient clarity of the cue regarding the consumption of the food, as no actual consumption-related cues were present. Therefore, in Study 3, we will introduce more explicit visual cues related to actual food consumption to further explore the impact of anthropomorphism strategies on food perception and consumption when controlling for the aesthetic factors of food aesthetic appeal during tasting scenarios.

## 5. Study 3

Study 3 was designed to directly test H2 by creating a clearer distinction between pre- and post-“consumption” within a controlled, simulated environment. It aimed to investigate whether making the act of consumption more explicit (via images of someone eating) would lead to a negative shift in evaluations specifically for anthropomorphic food after the simulation while holding aesthetic levels constant across food types.

### 5.1. Study Design

This study employed a mixed experimental design, wherein the between-subjects variable was the food type (anthropomorphic vs. non-anthropomorphic) and the within-subjects variable was the consumption type (pre-consumption vs. post-consumption). The dependent variables included gustatory perception, eating intention, and purchasing intention.

### 5.2. Participants

We collected data from a total of 80 participants. All participants were university students aged between 18 and 28 years (30 males; *M_age_* = 20.60, *SD_age_* = 1.83), and they received compensation for their participation. All procedures were sanctioned by the Institutional Review Board, and informed consent was obtained from all participants.

### 5.3. Food Materials

**Figure 7:**
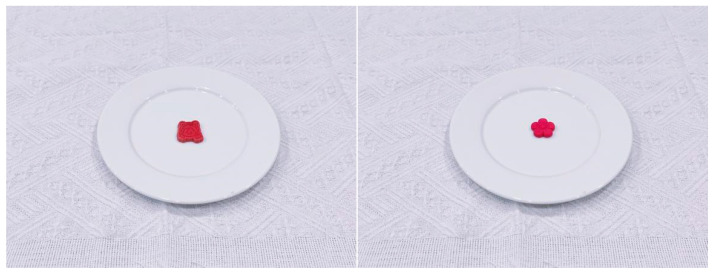
Cookies with different shapes but identical taste: (**left**) anthropomorphic cookie; (**right**) non-anthropomorphic cookie.

The study prepared two types of cookies (differing in shape but identical in taste: anthropomorphic shape vs. non-anthropomorphic shape, as shown in Figure 7). Prior to the main experiment, images of both types of cookies were taken with consistent pixel dimensions of 300 × 300. Subsequently, 38 participants (30 males; *M_age_* = 20.61, *SD_age_* = 2.70) who did not take part in the main experiment were recruited to evaluate the experimental materials. They were asked to assess the aesthetic appeal of the food presented in the images. The results indicated no significant differences between the anthropomorphic and non-anthropomorphic groups in terms of aesthetic appeal (*M_Anthropomorphic_* = 5.89, *SD_Anthropomorphic_* = 1.57 vs. *M_Non-anthropomorphic_* = 5.63, *SD_Non-anthropomorphic_* = 1.53, *t*(37) = 1.22, *p* = 0.23), thus supporting the validity of the material selection.

**Figure 8:**
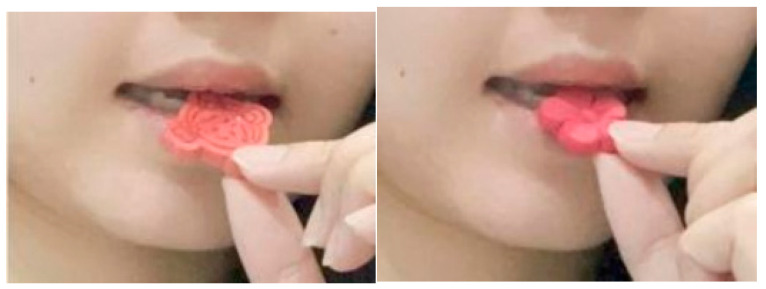
Images depicting simulated eating behavior.

Subsequently, a volunteer was recruited to partake in a real cookie tasting session. Her actual eating process was recorded, and the images from this session were utilized as visual cues for the authentic tasting in the subsequent experiment (see Figure 8).

### 5.4. Procedure

In this study, participants were recruited through an online platform and randomly assigned to questionnaire groups containing different food images. Prior to the experiment, participants completed a preliminary questionnaire including gender, age, eating intention, and hunger level. Participants then viewed an image of a cookie (see Figure 7) and were asked to observe and describe the image in detail.

Next, participants used a 7-point Likert scale to evaluate the cookie in terms of anthropomorphism, expected gustatory perception, eating intention, and purchasing intention. Subsequently, participants viewed simulated eating images and, following the instructions, imagined and mimicked the eating process to simulate tasting the cookie (see Figure 8).

After completing the simulated eating task, participants again used a 7-point Likert scale to evaluate the cookie in terms of post-consumption gustatory perception, eating intention, and purchasing intention.

### 5.5. Results

***Manipulation Check***. We conducted an independent samples *t*-test to compare the level of anthropomorphism between the anthropomorphic and non-anthropomorphic groups. The results showed that participants perceived the anthropomorphic group (*M_anthropomorphic_* = 6.23, *SD_anthropomorphic_* = 1.20) as significantly more human-like than the non-anthropomorphic group (*M_Non-anthropomorphic_* = 1.24, *SD_Non-anthropomorphic_* = 0.54), with *t*(78) = 24.15, *p* < 0.001, *Cohen’s d* = 5.36, indicating that our anthropomorphism manipulation was effective.

***Hunger level and baseline eating intention.*** We used independent samples *t*-tests to compare differences in hunger level and baseline eating intention between the anthropomorphic and non-anthropomorphic groups. The results showed no significant differences in hunger level nor baseline eating intention between the two groups (*ps*. > 0.05; see Table 5).

Subsequently, this study used Repeated Measures ANOVA to compare the effects of food type on gustatory perception, eating intention, and purchasing intention before and after consumption.

***Gustatory Perception.*** The main effect of food type was not significant, with *F*(1, 78) = 2.41, *p* = 0.12; the main effect of consumption type was also not significant, with *F*(1, 78) = 0.86, *p* = 0.36; and their interaction effect was not significant either, with *F*(1, 78) = 0.75, *p* = 0.39 (see Table 5).

**Figure 9:**
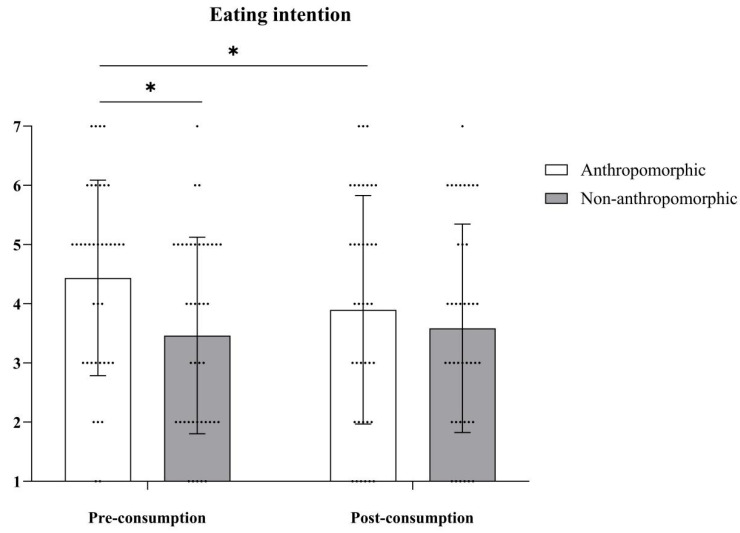
The effects of food type and consumption type on eating intention. The dots in the figure represent each participant’s data, the error bars indicate the standard error, and the asterisks denote statistical significance at *p* < 0.05.

***Eating Intention.*** The main effect of food type was not significant, with *F*(1, 78) = 2.97, *p* = 0.09. The main effect of consumption type was also not significant, with *F*(1, 78) = 2.94, *p* = 0.09. However, the interaction effect between food type and consumption type was significant, with *F*(1, 78) = 7.38, *p* = 0.008, *η_p_*^2^ = 0.09 (see Table 6 and Figure 9). Further simple effects analysis revealed that in the anthropomorphic group, eating intention before consumption (*M_Pre-consumption_* = 4.44, *SD_Pre-consumption_* = 1.65) was significantly higher than after consumption (*M_Post-consumption_* = 3.90, *SD_Post-consumption_* = 1.93), with *F*(1, 78) = 9.58, *p* = 0.003, *η_p_*^2^ = 0.11. In the non-anthropomorphic group, there were no significant differences in eating intention before and after consumption (*M_Pre-consumption_* = 3.46, *SD_Pre-consumption_* = 1.66; *M_Post-consumption_* = 3.59, *SD_Post-consumption_* = 1.76, *F*(1, 78) = 0.52, *p* = 0.47).

**Figure 10:**
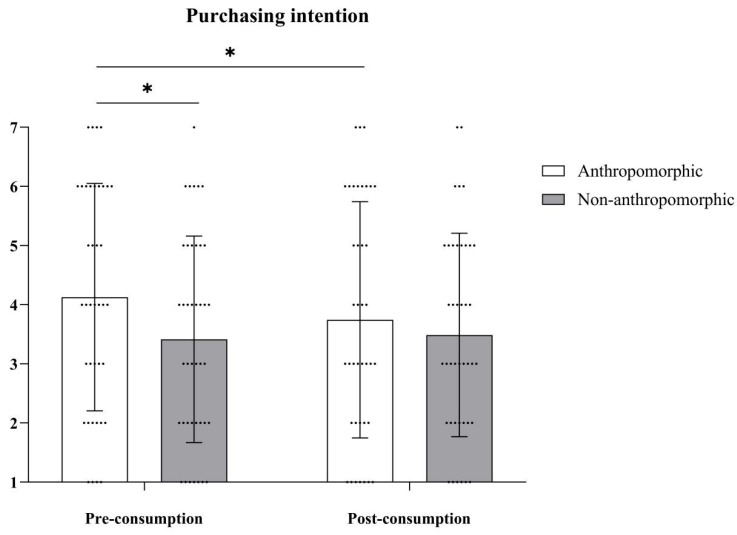
The effects of food type and consumption type on purchasing intention. The dots in the figure represent each participant’s data, the error bars indicate the standard error, and the asterisks denote statistical significance at *p* < 0.05.

***Purchasing Intention.*** The main effect of food type was not significant, with *F*(1, 78) = 1.47, *p* = 0.23; the main effect of consumption type was also not significant, with *F*(1, 78) = 2.29, *p* = 0.13. However, the interaction effect between food type and consumption type was significant, with *F*(1, 78) = 4.95, *p* = 0.03, *η_p_*^2^ = 0.06 (see Table 4 and Figure 10). Further simple effects analysis revealed that in the anthropomorphic group, purchasing intention before consumption (*M_Pre-consumption_* = 4.13, *SD_Pre-consumption_* = 1.92) was significantly higher than after consumption (*M_Post-consumption_* = 3.74, *SD_Post-consumption_* = 2.00), with *F*(1, 78) = 6.82, *p* = 0.01, *η_p_*^2^ = 0.08. In the non-anthropomorphic group, there was no significant difference in purchasing intention before and after consumption (*M_Pre-consumption_* = 3.41, *SD_Pre-consumption_* = 1.75; *M_Post-consumption_* = 3.49, *SD_Post-consumption_* = 1.72, *F*(1, 78) = 0.26, *p* = 0.61).

The results of Study 3 indicate that after controlling for aesthetic levels, the anthropomorphism strategy alters individuals’ eating intentions and purchasing intentions both before and after food consumption. Compared with non-anthropomorphic food, the consumption of anthropomorphic food resulted in a reduction in participants’ eating intentions and purchasing intentions. However, Study 3 primarily employed images to induce simulated eating behavior, which contradicts Epley’s perspective [38]. Therefore, in Study 4, we will aim to further investigate the impact of anthropomorphism on food perception and consumption during the actual tasting phase. Additionally, to assess the persistence of these effects, data will be collected again one week after the experiment to examine the influence of the anthropomorphism strategy on taste perception in repeated evaluation contexts.

## 6. Study 4

### 6.1. Study Design

This experiment employed a mixed experimental design, wherein the between-subjects variable was food type (anthropomorphic vs. non-anthropomorphic) and the within-subjects variable was consumption type (pre-consumption and post-consumption). The dependent variables included gustatory perception, eating intention, and purchasing intention.

### 6.2. Participants

We collected data from a total of 111 participants. All participants were university students aged between 18 and 28 years (16 males; *M_age_* = 20.51, *SD_age_* = 1.95). Participants received compensation for their participation. All procedures were sanctioned by the Institutional Review Board, and written informed consent was obtained from all participants.

### 6.3. Food Materials

Consistently with Study 3, we prepared two types of cookies (differing in shape but identical in taste: anthropomorphic shape vs. non-anthropomorphic shape, as shown in Figure 7). However, unlike Study 3, Study 4 utilized real cookies, requiring participants to engage in an actual eating task.

### 6.4. Procedure

During the experiment, we ensured that each session included two participants simultaneously. By pairing participants under different conditions and conducting the experiment at the same time, we controlled for hunger levels and any other time-related effects. Upon arrival, participants were randomly assigned to one of the two experimental conditions (anthropomorphic/non-anthropomorphic). Before entering the laboratory, participants completed a survey on demographic information (gender and age), eating intention, and hunger rating.

Participants were then directed to their assigned laboratory, where they were asked to sit and observe the food—cookies—on the table without being informed of the cookie type. First, participants were asked to observe the cookies and describe their specific shapes. Following this, they were asked to use a 7-point Likert scale to evaluate the cookies before consumption, including anthropomorphism (“This cookie design looks like a person,” 1 = strongly disagree to 7 = strongly agree), expected gustatory perception (pre-consumption gustatory perception, 1 = not tasty at all to 7 = very tasty), eating intention (pre-consumption eating intention, 1 = not at all to 7 = very much), and purchasing intention (pre-consumption purchasing intention, 1 = not at all to 7 = very much).

Subsequently, participants were asked to taste the cookies in front of them and then use the same 7-point Likert scale to evaluate the cookies post-consumption. This evaluation included gustatory perception (post-consumption gustatory perception, 1 = not tasty at all to 7 = very tasty), eating intention (post-consumption eating intention, 1 = not at all to 7 = very much), and purchasing intention (post-consumption purchasing intention, 1 = not at all to 7 = very much).

One week later, we distributed a follow-up survey to assess participants’ attitudes and consumption tendencies for anthropomorphic food after having consumed different types of cookies. In the survey, participants first completed demographic information and baseline ratings for eating intention and hunger level. They were then asked to observe an image of an anthropomorphic cookie (as shown in Figure 8, left image) and describe the anthropomorphic cookie presented. Subsequently, participants used the same 7-point Likert scale from the previous week to evaluate the presented cookie, including expected gustatory perception, eating intention, and purchasing intention.

### 6.5. Results

***Manipulation Check.*** We conducted an independent samples *t*-test to compare the level of anthropomorphism between the anthropomorphic and non-anthropomorphic groups. The results showed that participants perceived the anthropomorphic group (*M*_anthropomorphic_ = 6.36, *SD_anthropomorphic_* = 1.08) as significantly more human-like compared with the non-anthropomorphic group (*M*_Non-anthropomorphic_ = 1.18, *SD_Non-anthropomorphic_* = 0.47), with *t*(109) = 32.94, *p* < 0.001, *Cohen’s d* = 6.31, demonstrating the effectiveness of our anthropomorphic manipulation.

***Baseline eating intention and hunger level.*** We used independent samples *t*-tests to compare differences in baseline eating intention and hunger level between the anthropomorphic and non-anthropomorphic groups. The results indicated no significant differences in baseline eating intention or hunger level between the two groups (*ps.* > 0.05; see Table 7).

Subsequently, this study used Repeated Measures ANOVA to compare the effects of food type on gustatory perception, eating intention, and purchasing intention before and after consumption.

**Figure 11:**
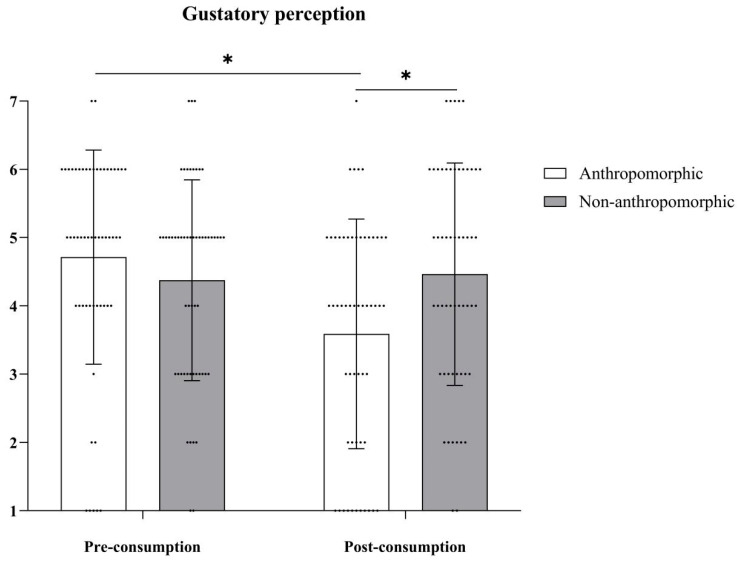
The effects of food type and consumption type on gustatory perception. The dots in the figure represent each participant’s data, the error bars indicate the standard error, and the asterisks denote statistical significance at *p* < 0.05.

***Gustatory Perception.*** The main effect of food type was not significant, with *F*(1, 109) = 0.70, *p* = 0.41; the main effect of consumption type was significant, with *F*(1, 109) = 11.19, *p* = 0.001, *η_p_*^2^ = 0.09; and their interaction effect was significant, with *F*(1, 109) = 15.30, *p* < 0.001, *η_p_*^2^ = 0.12 (see Table 8 and Figure 11). Further simple effects analysis revealed that in the anthropomorphic group, gustatory perception before consumption (*M_Pre-consumption_* = 4.78, *SD_Pre-consumption_* = 1.50) was significantly higher than after consumption (*M_Post-consumption_* = 3.64, *SD_Post-consumption_* = 1.66), with *F*(1, 109) = 26.10, *p* < 0.001, *η_p_*^2^ = 0.19. In the non-anthropomorphic group, there were no significant differences in gustatory perception before and after consumption (*M_Pre-consumption_* = 4.38, *SD_Pre-consumption_* = 1.47; *M_Post-consumption_* = 4.46, *SD_Post-consumption_* = 1.63, *F*(1, 109) = 0.16, *p* = 0.69).

**Figure 12:**
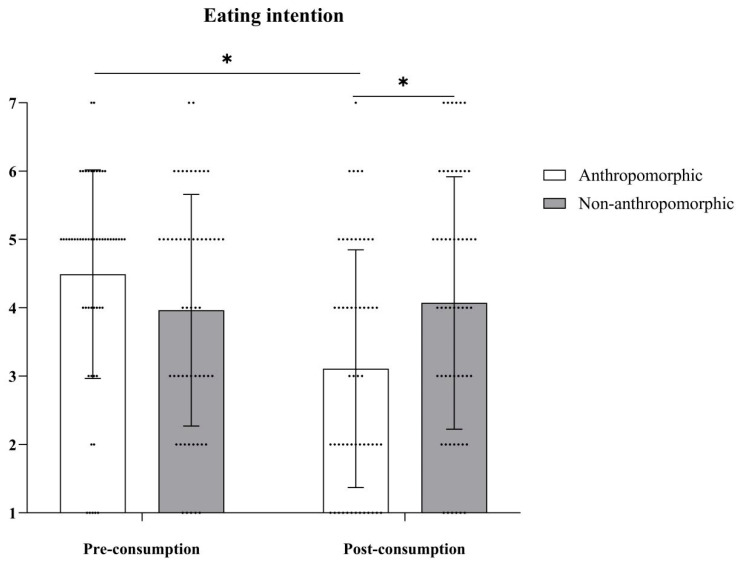
The effects of food type and consumption type on eating intention. The dots in the figure represent each participant’s data, the error bars indicate the standard error, and the asterisks denote statistical significance at *p* < 0.05.

***Eating Intention.*** The main effect of food type was not significant, with *F*(1, 109) = 0.64, *p* = 0.43; the main effect of consumption type was significant, with *F*(1, 109) = 13.27, *p* < 0.001, *η_p_^2^* = 0.11. The interaction effect between food type and consumption type was significant, with *F*(1, 109) = 18.11, *p* < 0.001, *η_p_*^2^ = 0.14 (see Table 6 and Figure 12). Further simple effects analysis revealed that in the anthropomorphic group, eating intention before consumption (*M_Pre-consumption_* = 4.49, *SD_Pre-consumption_* = 1.53) was significantly higher than after consumption (*M_Post-consumption_* = 3.11, *SD_Post-consumption_* = 1.74), with *F*(1, 109) = 30.91, *p* < 0.001, *η_p_*^2^ = 0.22. In the non-anthropomorphic group, there were no significant differences in eating intention before and after consumption (*M_Pre-consumption_* = 3.96, *SD_Pre-consumption_* = 1.69; *M_Post-consumption_* = 4.07, *SD_Post-consumption_* = 1.85, *F*(1, 109) = 0.19, *p* = 0.66).

**Figure 13:**
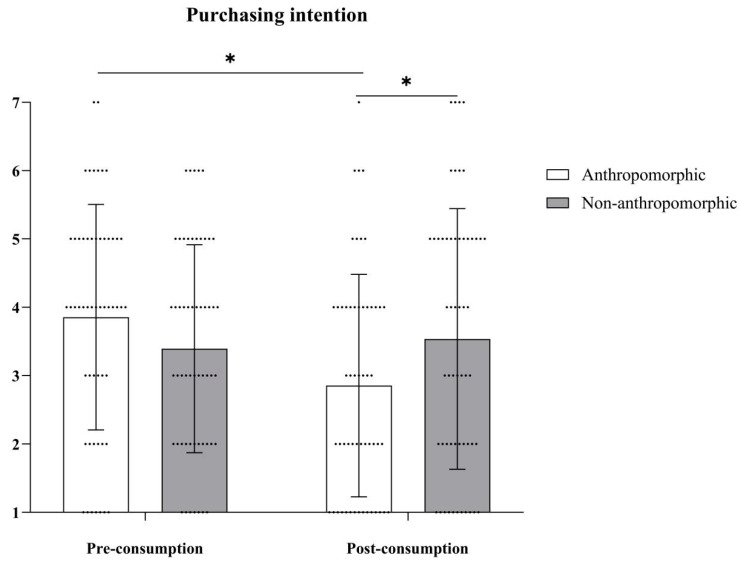
The effects of food type and consumption type on purchasing intention. The dots in the figure represent each participant’s data, the error bars indicate the standard error, and the asterisks denote statistical significance at *p* < 0.05.

***Purchasing Intention.*** The main effect of food type was not significant, with *F*(1, 109) = 0.15, *p* = 0.70; the main effect of consumption type was significant, with *F*(1, 109) = 8.52, *p* = 0.004, *η_p_*^2^ = 0.07. The interaction effect between food type and consumption type was significant, with *F*(1, 109) = 15.14, *p* < 0.001, *η_p_*^2^ = 0.12 (see Table 6 and Figure 13). Further simple effects analysis revealed that in the anthropomorphic group, purchasing intention before consumption (*M_Pre-consumption_* = 3.86, *SD_Pre-consumption_* = 1.65) was significantly higher than after consumption (*M_Post-consumption_* = 2.86, *SD_Post-consumption_* = 1.63), with *F*(1, 109) = 22.98, *p* < 0.001, *η_p_*^2^ = 0.17. In the non-anthropomorphic group, there were no significant differences in purchasing intention before and after consumption (*M_Pre-consumption_* = 3.39, *SD_Pre-consumption_* = 1.52; *M_Post-consumption_* = 3.54, *SD_Post-consumption_* = 1.91, *F*(1, 109) = 0.48, *p* = 0.49).

## 7. Repeated Evaluation

Due to 20 participants not participating in the follow-up survey one week later, these 20 participants were excluded from the statistical analysis. Therefore, the data from 91 participants (82 females, *M_age_* = 20.48, *SD_age_* = 1.92) were retained for analysis.

**Figure 14:**
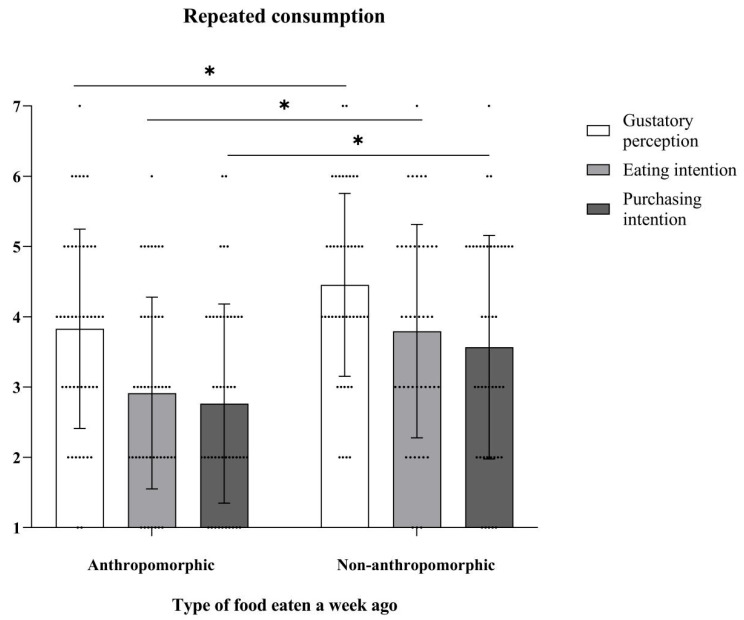
The effects of food type on consumer attitudes and consumption tendencies toward anthropomorphic food one week later (repeated evaluation). The dots in the figure represent each participant’s data, the error bars indicate the standard error, and the asterisks denote statistical significance at *p* < 0.05.

The follow-up data collected one week later showed that compared with the non-anthropomorphic group, participants who had consumed anthropomorphic cookies exhibited significantly lower gustatory perception, eating intention, and purchasing intention towards anthropomorphic cookies (*ps*. < 0.05; see Table 9 and Figure 14).

The results of Study 4 support H2 and H3, revealing that in a real tasting context, after controlling for the aesthetic appeal of the food, consumers exhibited a significant decrease in their gustatory expectations, eating intentions, and purchasing intentions for anthropomorphic food after consumption (vs. before consumption). Moreover, these negative effects persisted in the follow-up survey conducted one week later, where consumers’ evaluations of the food in a repeated evaluation context were more negative than during the initial consumption. This suggests that anthropomorphic food exerts a lasting negative impact on continued food perception and consumption.

## 8. General Discussion

In the present research study, we systematically examined the effects of anthropomorphism on consumers’ perceptions of food across four studies, while controlling for the aesthetic appeal of the food. First, the results of Study 1 were consistent with our expectations. In a context involving only the choice stage, both the aesthetic appeal of food and the anthropomorphic strategies exerted significant positive effects on consumers’ gustatory perceptions and eating intentions. The findings regarding purchasing intentions were consistent with those reported by Cooremans and Geuens [10], suggesting that anthropomorphism can serve as a means of alleviating the negative effects associated with low aesthetic appeal. These results indicate that at the choice stage, both aesthetic factors and anthropomorphic strategies positively influence consumers’ perceptions of food.

In Study 2, we varied the materials and introduced a relatively subtle cue indicating that the food would be eaten (e.g., an image of a hand holding chopsticks). The results did not fully support Hypothesis 2, as the negative effect of anthropomorphism was not significant. A possible explanation is that the “food will be eaten” cue in Study 2 was not sufficiently salient for consumers to be fully aware of the impending act.

In Study 3, we further introduced explicit cues signaling real food consumption. When aesthetic appeal was controlled, participants’ eating and purchasing intentions for anthropomorphized foods significantly decreased both before and after simulated consumption, supporting Hypothesis 2. However, no significant differences emerged in gustatory perception evaluations; anthropomorphized foods did not exhibit a significant negative effect either before or after the simulated consumption, perhaps because it did not fully approximate actual eating, and participants may not have fully realized that they would indeed consume the anthropomorphized food in reality.

Therefore, in Study 4, we employed an actual tasting task and examined the effects of anthropomorphized foods on consumers in a repeated evaluation context while controlling for aesthetic appeal. The results showed that during the initial tasting, participants’ ratings of gustatory perception and eating and purchasing intentions regarding the anthropomorphized foods were significantly lower both before and after consumption, supporting Hypothesis 2. These findings indicate that once consumers are placed in a context in which they actually consume anthropomorphized food, it exerts a negative effect on taste perception. This result is consistent with prior research documenting the negative post-consumption effects of anthropomorphized foods [15,39]. Furthermore, in a follow-up session conducted one week after the initial consumption, participants who engaged in repeated consumption provided significantly more negative evaluations of gustatory perception and eating and purchasing intentions than those who had initially consumed non-anthropomorphized food. This finding supports Hypothesis 3 and corroborates Schroll et al.’s [15] proposition that anthropomorphism can lead to negative effects in repeated evaluation contexts.

Overall, this study is the first to systematically explore the potential relationship between anthropomorphic strategies and the aesthetic appeal of food in shaping taste perception. From a theoretical perspective, the results indicate that when aesthetic influences are controlled, anthropomorphism only exerts a positive effect on taste expectations at the choice stage, namely, when consumers are not yet aware that they will actually consume the anthropomorphized food. However, as consumers become increasingly aware that the food will be eaten, the positive effect of anthropomorphism diminishes; once consumers actually consume the food, anthropomorphic strategies instead have a negative impact on taste perception, and this effect persists over time. These findings offer a novel theoretical interpretation of anthropomorphic strategies, suggesting that in many prior studies that did not control for aesthetic factors, the observed positive effects of anthropomorphism in actual tasting contexts were likely driven by unintended enhancements in the food’s aesthetic appeal rather than by anthropomorphism itself.

From a practical perspective, anthropomorphism has long been an important strategy closely associated with food marketing practices. From the beloved Mickey Mouse character at Disneyland to anthropomorphized candies sold by street vendors, these strategies have been applied across a wide range of food consumption contexts. With the advent of the experience economy, consumers increasingly seek memorable consumption experiences and more enjoyable dining experiences, with aesthetic factors playing a critical role in these pursuits [40,41]. Thus, examining the effects of anthropomorphism while controlling for aesthetic appeal has important practical implications. Notably, our findings suggest that in today’s context—where consumers continuously pursue more pleasurable food experiences—anthropomorphism may not always be an optimal marketing strategy and may, in some situations, undermine consumers’ perceptions of a food’s tastiness.

## 9. Limitations and Future Studies

This study has some limitations at the operational level. First, only two types of food—cookies and bento—were used, with only cookies being employed in the actual tasting stage, suggesting that future research should incorporate a wider variety of food items. Furthermore, Study 4 involved actual tasting in a laboratory setting, which may raise concerns regarding ecological validity. Additionally, when distinguishing between the consumer selection phase and the actual tasting phase, no fine-grained differentiation was made in terms of intensity. Although Study 2 included a cue image involving hands and chopsticks to signify eating and Study 3 featured realistic photos of simulated consumption, neither clearly distinguished the extent to which participants perceived “actually eating anthropomorphized food,” which may explain why some results did not align with the hypotheses. In addition, this study also has certain limitations in terms of sample size. Specifically, the sample sizes in Study 3 were relatively small, which may have resulted in insufficient statistical power. Additionally, although we aimed to explore the impact of anthropomorphized food on consumer evaluations in repeated consumption scenarios through Study 4, the fact that participants did not consume the food a second time but merely repeated their evaluations of the food is a limitation of this study. It should be noted that the participants in this study were recruited solely from a university and were all young adults. Such an age distribution may impose certain limitations on the generalizability of our research findings.

As a consequence, the findings are more susceptible to individual differences, and the stability and replicability of the results may be limited. Lastly, in this study, we only employed a one-week follow-up for repeated measurements, without considering the more long-term impact of anthropomorphized food on consumers’ future eating behaviors.

## 10. Conclusions

In summary, in this study, we reveal that the influence of anthropomorphism on food taste evaluation varies across different stages. During the food selection phase, anthropomorphism has a positive effect on taste expectations, which does not interact with the aesthetic appeal of the food. However, in actual consumption situations, after controlling for the food’s aesthetic appeal, anthropomorphism may have a negative impact on taste evaluation. Furthermore, the results of this study suggest that when controlling for aesthetic appeal, anthropomorphism may reduce consumers’ taste evaluations in repeated evaluation contexts. The findings provide new empirical evidence and theoretical support for understanding the influence of anthropomorphism on perceptions of food taste.

## Figures and Tables

**Table 1:** The aesthetic appeal of four different types of cookies (*M* ± *SD*).

		Aesthetic Appeal	
		High	Low
**Food type**	Anthropomorphic	6.02 ± 1.91	5.29 ± 2.17
	Non-anthropomorphic	5.82 ± 1.70	5.14 ± 1.80

**Table 2:** The aesthetic appeal of four different types of bento (*M* ± *SD*).

		Aesthetic Appeal	
		High	Low
**Food type**	Anthropomorphic	5.51 ± 1.20	3.83 ± 1.40
	Non-anthropomorphic	4.95 ± 1.41	3.85 ± 1.51

**Table 3:** The Anthropomorphism Rating of four different types of bento (*M* ± *SD*).

		Aesthetic Appeal	
		High	Low
**Food type**	Anthropomorphic	5.11 ± 1.11	4.86 ± 1.34
	Non-anthropomorphic	1.60 ± 1.13	1.85 ± 1.34

**Table 4:** The hunger level of four different types of foods (*M* ± *SD*).

		Aesthetic Appeal	
		High	Low
**Food type**	Anthropomorphic	5.32 ± 1.78	5.10 ± 2.07
	Non-anthropomorphic	5.46 ± 2.24	5.51 ± 2.02

**Table 5:** The mean scores of different foods.

	Food Type		*t*	*p*	95%CI	
	Anthropomorphic	Non-Anthropomorphic			Lower	Upper
**Hunger level**	5.51 ± 2.29	6.07 ± 1.63	−1.26	0.21	−1.44	0.32
**Eating intention**	5.21 ± 2.25	4.63 ± 2.10	1.18	0.24	−0.40	1.54

**Table 6:** The mean scores of different foods.

		Food Type		*t*	*p*	95%CI		*Cohen’s d*
		Anthropomorphic	Non-Anthropomorphic			Lower	Upper	
**Pre-consumption**	Gustatory perception	4.72 ± 1.40	4.32 ± 1.46	1.26	0.21	−0.23	1.04	
	**Eating intention**	**4.44 ± 1.65**	**3.46 ± 1.66**	**2.63**	**0.01**	**0.24**	**1.71**	**0.60**
	Purchasing intention	4.13 ± 1.92	3.41 ± 1.75	1.74	0.09	−0.10	1.53	
**Post-consumption**	Gustatory perception	4.46 ± 1.59	4.24 ± 1.79	0.58	0.57	−0.54	0.97	
	Eating intention	3.90 ± 1.93	3.59 ± 1.76	0.76	0.45	−0.51	1.13	
	Purchasing intention	3.74 ± 2.00	3.49 ± 1.72	0.62	0.54	−0.57	1.08	

**Table 7:** The mean scores of different foods.

	Food Type		*t*	*p*	95%CI	
	Anthropomorphic	Non-Anthropomorphic			Lower	Upper
**Hunger level**	5.67 ± 1.98	5.98 ± 1.93	−0.83	0.41	−1.05	0.43
**Eating intention**	4.62 ± 1.78	4.27 ± 2.13	0.94	0.35	−0.39	1.09

**Table 8:** The mean scores of different foods.

		Food Type		*t*	*p*	95%CI		*Cohen’* *s d*
		Anthropomorphic	Non-Anthropomorphic			Lower	Upper	
**Pre-consumption**	Gustatory perception	4.78 ± 1.50	4.38 ± 1.47	1.44	0.15	−0.15	0.97	
	Eating intention	4.49 ± 1.53	3.96 ± 1.69	1.72	0.09	−0.08	1.13	
	Purchasing intention	3.86 ± 1.65	3.39 ± 1.52	1.53	0.13	−0.14	1.06	
**Post-consumption**	**Gustatory perception**	**3.64 ± 1.66**	**4.46 ± 1.63**	**−2.65**	**0.01**	**−1.45**	**−0.21**	**−0.51**
	**Eating intention**	**3.11 ± 1.74**	**4.07 ± 1.85**	**−2.83**	**0.01**	**−1.64**	**−0.29**	**−0.54**
	**Purchasing intention**	**2.86 ± 1.63**	**3.54 ± 1.91**	**−2.02**	**0.05**	**−1.35**	**−0.01**	**−0.39**

**Table 9:** The mean scores of different foods.

	Food Type		*t*	*p*	95%CI		*Cohen’s d*
	Anthropomorphic	Non-Anthropomorphic			Lower	Upper	
**Gustatory perception**	3.83 ± 1.42	4.46 ± 1.30	−2.18	0.03	−1.19	−0.06	−0.46
**Eating intention**	2.92 ± 1.36	3.80 ± 1.52	−2.91	0.01	−1.48	−0.28	−0.62
**Purchasing intention**	2.77 ± 1.42	3.57 ± 1.59	−2.54	0.01	−1.43	−0.18	−0.54

## Data Availability

The original contributions presented in the study are included in the article. Further inquiries can be directed to the corresponding authors.
